# The choroid plexus is modulated by various peripheral stimuli: implications to diseases of the central nervous system

**DOI:** 10.3389/fncel.2015.00136

**Published:** 2015-04-13

**Authors:** Fernanda Marques, João C. Sousa

**Affiliations:** ^1^Life and Health Sciences Research Institute (ICVS), School of Health Sciences, University of MinhoBraga, Portugal; ^2^ICVS/3B’s - PT Government Associate LaboratoryBraga/Guimarães, Portugal

**Keywords:** choroid plexus, epithelial cell, lipocalin 2, inflammation, multiple sclerosis, experimental autoimmune encephalomyelitis, astrocytes, cerebrospinal fluid

## Abstract

The blood brain barrier (BBB) and the blood cerebrospinal fluid barrier (BCSFB) form the barriers of the brain. These barriers are essential not only for the protection of the brain, but also in regulating the exchange of cells and molecules in and out of the brain. The choroid plexus (CP) epithelial cells and the arachnoid membrane form the BCSFB. The CP is structurally divided into two independent compartments: one formed by a unique and continuous line of epithelial cells that rest upon a basal lamina; and, a second consisting of a central core formed by connective and highly vascularized tissue populated by diverse cell types (fibroblasts, macrophages and dendritic cells). Here, we review how the CP transcriptome and secretome vary depending on the nature and duration of the stimuli to which the CP is exposed. Specifically, when the peripheral stimulation is acute the CP response is rapid, strong and transient, whereas if the stimulation is sustained in time the CP response persists but it is weaker. Furthermore, not all of the epithelium responds at the same time to peripheral stimulation, suggesting the existence of a synchrony system between individual CP epithelial cells.

## Introduction

The brain is an unusual tissue since it is protected from free exchange with the blood. This is attained via the presence of blood-brain barriers. Two main barriers exist: the blood-brain barrier (BBB) and the blood-cerebrospinal fluid (CSF) barrier (BCSFB; Abbott, 2005). The presence of these barriers contributes, in part, to the central nervous system (CNS) homeostasis. It was generally accepted that under normal conditions, the BBB and the BCSFB prevent immune cell entry into the CNS. This view has changed since it is now clearly recognized that activated T cells are able to cross the brain barriers to perform immune surveillance of the CNS (Engelhardt and Coisne, 2011).

In this review, we will focus on the BCSFB, located at the choroid plexus (CP) level, particularly on (i) how the BCSFB provides signals into the brain, in conditions of peripheral and central inflammation; and on (ii) the specific response of individual CP epithelial cells.

## Choroid Plexus Morphology and Functions

At the CP level, the BCSFB is composed by a monolayer of epithelial cells that contain tight junctions in the lateral wall of the CP epithelial cells. This epithelial layer rests in a basal lamina surrounding and enclosing a central stroma where dendritic cells, fibroblasts and macrophages can be found. The blood vasculature that irrigates the CP central stroma is fenestrated; thus, the cellular movement of molecules and cells within the CP stroma is not restricted. Ultrastructurally, the epithelial cells contain a high density of mitochondria, Golgi apparatus and a smooth endoplasmic reticulum, therefore demonstrating highly synthetic and secretory capacity. This is necessary given the CP principal function: the production of CSF (Speake et al., 2001). The CSF is vital for normal brain function, ensuring an optimal environment for neuronal cells. More so, a high number of vesicles with lysosomal characteristics are present in the CP cytoplasm. Attached to the apical side of the CP epithelial cells, the epiplexus cells [also known as Kolmer cells (Pietzsch-Rohrschneider, 1980)], which present macrophage activity, survey the CSF filled brain ventricles. In addition to these cells, free floating and supraependymal cells are also observed in the lumen of the ventricles (Matyszak et al., 1992; McMenamin et al., 2003; Nataf et al., 2006). These cells, altogether referred as intraventricular macrophages, are likely to represent the first line of defense capable to react in brain infections, after bacteria transverse the CP.

Interestingly, depending on the pathological conditions, the composition of the stromal compartment is highly changeable, and may include other peripheral immune cells such as neutrophils, monocytes, T and B cells (Marques et al., 2012; Schmitt et al., 2012; Szmydynger-Chodobska et al., 2012). Necessarily, when addressing the cellular contribution for the overall CP response, two CP compartments should be considered: (i) one related to the CP epithelial cells, which directly influences the composition of the CSF; and (ii) one other relative to the stromal cells. The latter, can also affect the CP epithelial cells secretome by secreting molecules that signal the epithelial cells. Finally, when addressing the CP response, it is also of relevance the type and duration of stimuli to which the CP is exposed. This will be next reviewed in the context of peripheral inflammation.

## Acute, Sustained and Attenuated Responses: Does the CP have an On/Off System?

How the brain barriers respond to peripheral stimuli, and how this response is conveyed into the brain, is of great relevance to understand normal brain homeostasis and diseases of the CNS. The presence of several receptors and transporters in the cells that constitute the brain barriers demonstrate that the barriers are not simple obstacles for molecules and cells but, also, active metabolic players. The observation that inflammation seems to be common to most brain disorders has raised interest on how inflammatory stimuli in the periphery may trigger events directly or indirectly related to brain pathology. Thus, studying how the barriers’ respond to specific inflammatory stimuli/mediators may help understand their function in disease. The CP response to peripheral inflammation, studied upon intraperitoneal injection of the Gram negative bacteria cell wall lipopolyssacharide (LPS) component, has revealed how differential the response may be, providing clues on how the communication between the periphery and the brain is established. Upon a single acute LPS injection, the CP responds steeply, with alterations in its transcriptome already detectable upon 1 h, peaking at 3–6 h and returning close to basal levels at 72 h (Marques et al., 2009a). Of interest, the expression level, in terms of fold change, observed for some genes is above 50 (Marques et al., 2009a). Interestingly, if the same stimulus is repeated every 2 weeks for a period of 3 months, the CP displays an attenuated response, with few genes presenting fold changes above 5 (Marques et al., 2009b). These studies indicate that in the repeated stimulation paradigm, the CP response is sustained in time, even 15 days after the last LPS injection (Marques et al., 2009b). Indeed, it seems that the repeated injection of LPS induces a sustained, albeit “milder”, transcription of specific genes encoding for molecules already found transiently altered upon a single LPS injection. Interestingly, in the relapse/remitting experimental autoimmune encephalomyelitis (EAE) animal model of multiple sclerosis (MS), a condition of continuous inflammation, the same attenuated response was been observed (Marques et al., 2012). Specifically, the expression of several genes is found altered in all phases of the disease, but with fold changes much smaller when compared to those observed after a single acute stimulus (Marques et al., 2009a).

Of interest, the observation regarding the attenuated CP response to the chronic vs. acute stimulus is not specific for the inflammatory challenges. In a recent study, the CP gene expression profile analysis of individuals with major depressive disorder (a chronic disease) showed that the genes most altered presented a maximum fold change of 3.5 times (Turner et al., 2014). In addition, a study with female and male rats 2 weeks after gonadectomy also suggested that chronic stimuli are associated with lower fold changes in the CP gene expression profile. Specifically, although a large number of differentially expressed genes were detected, the majority of the genes displayed a 1.5-fold change, when compared to sham controls, both for males and females (Quintela et al., 2013). Although it is not know whether the CP response would be different if the CP was analyzed in a shorter time point after the surgery, the authors observed that for females 1168 genes showed a fold increase of 1.5, 422 a fold increase of 2 and only 11 genes a fold increase of 5. The same scenario was found for the down-regulated genes. Altogether, these observations suggest that the CP adapts its response to the type of stimulus, with a weaker response under chronic stimulation. Of relevance this is not a specific response of the CP since it was also shown that the liver response to acute and chronic stimulus differs (Crispe et al., 2006).

## Two Compartments, Two Different Responses

**Figure 1:**
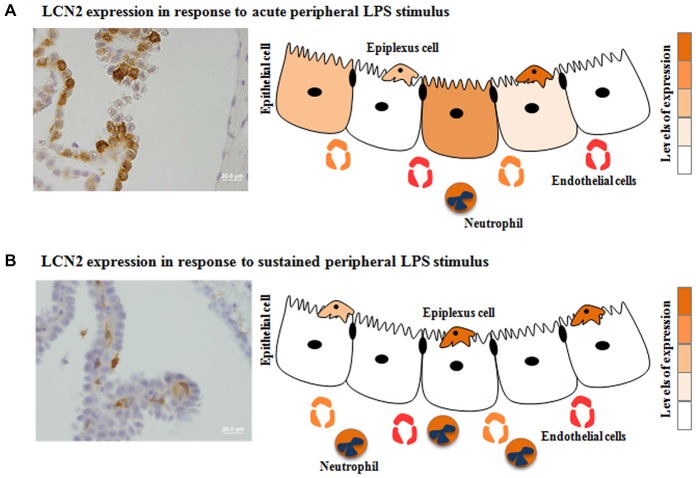
**Choroid plexus (CP) differential response to acute *vs*. sustained inflammation: the case for the lipocalin 2 (LCN2)**. The expression of certain CP proteins seems to be influenced by the duration of the stimulus: acute **(A)** or sustained **(B)** peripheral inflammation. Also even when a stimulus is acute, not all cells are producing the same protein. Herein is demonstrated the specific case for LCN2 in response to acute vs. sustained peripheral inflammation.

As previously mentioned, two compartments can be found at the CP level. This distinction is relevant when analyzing microarray data, since the majority of the studies describe the CP response as a whole, thus taking into account both epithelial and stromal cells. It may be necessary to discriminate both contributions, as they likely influence the brain differently. Alterations in the CP epithelia transcriptome may directly alter the CSF composition and the number of transporters and receptors in the cell membrane; while changes in the stroma cells transcriptome may result in the secretion of molecules that may interact with receptors and transporters in the basal membrane of the epithelial cells, indirectly influencing their function. This distinction is relevant when analyzing the CP response to inflammation, infection or even neurodegeneration, since the composition of the CP stroma is enriched with immune cells. In accordance, a protein known as lipocalin 2 (LCN2), a molecule able to bind iron-loaded bacterial siderophores, is rapidly produced by the CP epithelium when an acute inflammatory stimulus is induced in the periphery (Marques et al., 2008). However, when the inflammatory stimulus persists, it is present solely in neutrophils infiltrating the CP stroma (Marques et al., 2009b, 2012). These findings suggest that the CP epithelium shuts down (or attenuates) the response whenever the system is challenged with prolonged stimulation (Figure 1).

Of note, to exactly pinpoint the origin of particular changes, the contribution of individual cells, or cell types, must be determined by using methodologies such as laser microdissection (Janssen et al., 2013), *in situ* hybridization and/or high throughput single cell transcriptomics. For acute stimulus, the use of *in vitro* models of CP epithelial cells (Monnot and Zheng, 2013) might additionally allow a precise mechanistic dissection of specific signal transduction pathways.

## Are All CP Epithelial Cells Responding Similarly to a Stimulus?

The characteristic hallmark of the BBB and the BCSFB to prevent paracellular diffusion is the presence of intercellular junctions, tight junctions and adherens junctions (Kniesel and Wolburg, 2000; Vorbrodt and Dobrogowska, 2003), between endothelial cells of the brain capillaries (Pachter et al., 2003; Persidsky et al., 2006) and between epithelial cells of the CP (Szmydynger-Chodobska et al., 2007). Tight and adherens junctions are known as the principal constituents of the junctional complex; but, the existence of the transmembrane connexins proteins, as a third partner in the intercellular proteins, allows the formation of hemichannels that combine to form intercellular gap junctions. Although connexins co-exist within the junctional complex, their role in barrier function of the BBB and the BCSFB has been mostly ignored. Recently, and regarding the role of connexins in the brain barriers, it was shown that the intracellular calcium concentration is an important factor for the permeability of the BBB and that this permeability is dependent on the effect of spatially propagated intercellular calcium waves through connexins (De Bock et al., 2012). Also the relevance of connexins in the intercellular communication of calcium was shown to be relevant for endothelial regeneration at the wound site. Additionally connexins stimulation might trigger proliferative and migratory behaviors in endothelial cells facing the lesion site (Moccia et al., 2014). Specifically at the CP epithelium level the role of connexins in the communication and in the orchestration of the overall CP response to stimuli, as it happens in the BBB, has been neglected. This aspect is relevant given the fact that CP epithelial cells seem to display individual transcription patterns. In fact, it was observed that while all the epithelial layer is immunolabeled for the protein transthyretin [encoded by the gene mostly expressed by the CP epithelial cells (Sousa et al., 2007)], not all cells label for LCN2 in response to acute peripheral inflammation (Marques et al., 2008). Therefore, it seems that not all CP epithelial cells follow the same response pattern (Figure 1). In future studies, the use of high-throughput single-cell transcriptomics will determine the extent and regulation of gene expression variation between identical cells. It can, nonetheless, be postulated that CP epithelial cells communicate with each other, through connexins, and that the individual response of one epithelial cell may influence the response of the neighboring cell. Of notice, a recent study showed that preventing cell-to-cell communication substantially reduced variability between cells in the expression of a set of early inflammation-related genes (Shalek et al., 2014). This suggests that paracrine signaling additionally represses part of the inflammatory program (Shalek et al., 2014). Importantly, this cell-to-cell communication might control the cellular heterogeneity and contribute to the overall response of the system, which has not yet been addressed at the CP level. Another interesting possibility, for the heterogeneity in the response of individual CP epithelial cells, may be a differential distribution in the type and amount of receptors present in each cell. If this is the case, each CP epithelial cell would be differentially triggered by the stimuli. Of interest, the expression of connexins in the CP is also altered in various conditions. Namely, connexin 43 (Cx43) expression is increased during the development of acute EAE and decreased in the remission phase of the disease (Jovanova-Nesic et al., 2009). Also, the inner diameter of the gap junction channels decreases in the CP of acute EAE, as measured by atomic force microscopy (Jovanova-Nesic et al., 2009). However, the relevance of these observations is unclear and deserves to be better explored; to date little is known regarding the regulation of these connexins in the CP in other CNS pathologies.

## The CP is Activated When the Stimulation is from the Brain to the Periphery: Recent Relevant Findings

Inflammation in the CNS (termed neuroinflammation) is common to all neurodegenerative conditions and whenever an injury is imposed in the CNS. In the resolution of neuroinflammation both resident and infiltrating immune cells play a role. Concerning the recruitment of peripheral cells, and unlike the BBB, the CP constitutively expresses adhesion molecules and chemokines, which support the preferential and initial transepithelial leukocyte trafficking across the CP (Steffen et al., 1996; Kunis et al., 2013). Of interest, it was shown that in situations of brain inflammation, as is the case of traumatic brain injury, neutrophils reach the CSF through the CP and accumulate in the CSF space near the injury site, from where they may migrate to the brain parenchyma (Szmydynger-Chodobska et al., 2009). Similarly, it has been suggested that the initial migration of Th17 cells into the brain parenchyma in the EAE animal model (Reboldi et al., 2009) occurs at the CP, and that monocytes migrate through the CP on their way to the lesion site in a spinal cord injury model (Shechter et al., 2013). In addition, the entry of cells into the brain through the CP is likely due to the basal expression of adhesion molecules in the epithelial cells, and also to the enrichment of the CP stroma with CD4+ T cells specific for CNS antigens, even in basal conditions (Baruch and Schwartz, 2013; Kunis et al., 2013). A recent work showed that in normal aging the expression of interferon type I (IFN-I) dependent genes [intercellular adhesion molecule 1(IFN-β) and interferon regulatory factor 7 (IRF7)] is strongly increased in the CP; whereas, interferon type II (IFN-II) dependent genes [intercellular adhesion molecule 1 (ICAM1) and interferon gamma-induced protein 10 (Cxcl10)] expression is found decreased (Baruch et al., 2014). This may indicate a decrease in the immune cell recruitment to the brain at least through the CP (Baruch et al., 2014). In addition, this age-associated IFN-II expression program is modulated by both circulation and brain-derived factors, whereas that of IFN-I seems more likely to be influenced by CSF-born factors in the aged brain (Baruch et al., 2014).

Future studies should, therefore, not only study the CP expression profile in diseases such as Alzheimer’s disease (AD), but also the overall CP response to inflammatory stimulus elicited both in the circumventricular space and within the brain parenchyma. In such an approach it would also be interesting to evaluate the interaction between epithelial and dendritic cells in regulating antigen sensitization to the periphery. It has been shown in the intestine and in the airways epithelia that intraepithelial dendritic cells extend processes into the intestinal and the airway lumen, respectively, to collect antigenic material from the mucosa surface (Kato and Schleimer, 2007; Schleimer et al., 2007; Shaykhiev and Bals, 2007). Whether such interaction also occurs between the dendritic cells within the CP epithelium and the ventricular space of the brain is of great relevance in understanding CNS diseases. One other relevant future challenge is the potential role of the CP as a modulator of CNS disorders namely neurodegenerative diseases. Of relevance the results showing that molecules produced by the CP impact on brain function (such as cognition) and brain repair represent an important starting point to tackle neurodegeneration. Also the role of the CP as the initial entry gate for immune cells access to the brain might be used and modulated aiming to decrease neuroinflammation associated with diseases such as MS.

## Conflict of Interest Statement

The authors declare that the research was conducted in the absence of any commercial or financial relationships that could be construed as a potential conflict of interest.
